# The false dichotomy of surgical futility in the emergency laparotomy setting: scoping review

**DOI:** 10.1093/bjsopen/zrac023

**Published:** 2022-04-07

**Authors:** Hannah Javanmard-Emamghissi, Sonia Lockwood, Sarah Hare, Jon N. Lund, Gillian M. Tierney, Susan J. Moug

**Affiliations:** Faculty of Medicine, Division of Health Sciences and Graduate Entry Medicine, University of Nottingham at Derby, Royal Derby Hospital, Derby, UK; Department of Colorectal Surgery, Bradford Royal Infirmary, Bradford, UK; Department of Anaesthesia, Medway Maritime Hospital, Kent, UK; Faculty of Medicine, Division of Health Sciences and Graduate Entry Medicine, University of Nottingham at Derby, Royal Derby Hospital, Derby, UK; Department of Colorectal Surgery, Royal Derby Hospital, Derby, UK; Department of Colorectal Surgery, Royal Alexandra Hospital, Paisley, UK

## Abstract

**Background:**

Futile is defined as ‘the fact of having no effect or of achieving nothing’. Futility in medicine has been defined through seven guiding principles, which in the context of emergency surgery, have been relatively unexplored. This scoping review aimed to identify key concepts around surgical futility as it relates to emergency laparotomy.

**Methods:**

Using the Arksey and O’Malley framework, a scoping review was conducted. A search of the Cochrane Library, Google Scholar, MEDLINE, and Embase was performed up until 1 November 2021 to identify literature relevant to the topic of futility in emergency laparotomy.

**Results:**

Three cohort studies were included in the analysis. A total of 105 157 patients were included, with 1114 patients reported as futile. All studies were recent (2019 to 2020) and focused on the principle of quantitative futility (assessment of the probability of death after surgery) within a timeline after surgery: two defining futility as death within 48 hours of surgery and one as death within 72 hours. In all cases this was derived from a survival histogram. Predictors of defined futile procedures included age, level of independence prior to admission, surgical pathology, serum creatinine, arterial lactate, and pH.

**Conclusion:**

There remains a paucity of research defining, exploring, and analysing futile surgery in patients undergoing emergency laparotomy. With limited published work focusing on quantitative futility and the binary outcome of death, research is urgently needed to explore all principles of futility, including the wishes of patients and their families.

## Teaser

Futility in surgery is a contentious subject that has been relatively unexplored in the emergency laparotomy setting. This scoping review identified three studies that examined quantitative futility following emergency laparotomy. With limited published work focusing on quantitative futility and the binary outcome of death, research is urgently needed to explore all principles of futility, including the wishes of patients and their families.

## Introduction

Futile is defined as ‘the fact of having no effect or of achieving nothing’^1^. In medicine there has been discussion about the controversial concept of treatment futility, resulting in differing views on what constitutes futile treatment, including those that believe the term should not exist at all^2^.

**Fig. 1:**
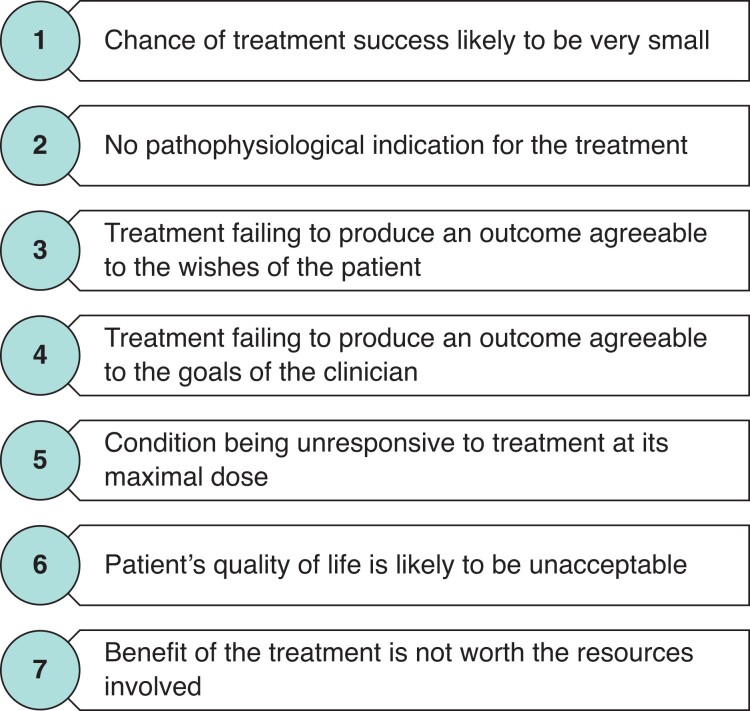
**The principles of futility in medical care** ^2^

In a 1995 book on medical ethics, Dr Bernard Lo described seven principles that defined futility in medical care: situations where the chance of treatment success was likely to be very small; no physiological indications for the treatment; condition is unresponsive to the treatment at its maximal dose; treatment is unlikely to produce an outcome that is in line with the wishes of the patient, goals of the clinician, or gives the patient an acceptable quality of life or the treatment is not worth the resources involved (*Fig. 1*)^3^. It is apparent that futility should be put in context, that not all principles are relevant in all cases, that some will overlap, and that none immediately outweigh another. As a result, there may be clinical settings where the relevant principles are in direct conflict with each other. Circumstances like that are demonstrated by recent high-profile legal cases such as that of Charlie Gard and Alfie Evans^4,5^.

With over 30 000 emergency laparotomies performed a year in the UK, surgeons and anaesthetists make daily complex decisions about whether to perform emergency surgery for patients whose comorbidities or functional status put them at high risk of adverse outcomes^6^. Decision making in the emergency laparotomy setting is hyperacute and multifaceted, involving rapid multidisciplinary communication and coordination with the patient and their relatives to reach shared decisions in their care. The National Emergency Laparotomy Audit (NELA) was established to define and subsequently improve outcomes for this large high-risk population^6^. Initially, the focus was reducing mortality and these population-based statistics have developed the NELA risk calculator, which provides guidance for the perioperative team as to the risk of death within 30 days^7^. Applying the principles, it can be seen that the NELA, although not explicitly defining the futile patient, applies the concept of quantitative futility (principle 5).

One of the challenges inherent to quantitative futility is the concept of ‘virtual certainty’^7^. Schneiderman and colleagues reframed the idea of ‘virtual certainty’ as an exceedingly low probability of success, arguing that ‘most of us probably would agree that if a treatment has not worked in the last 100 cases, almost certainly it is not going to work if it’s is tried again’^8,9^. Attempts to determine empirically the threshold for quantitative futility have resulted in responses between 0 per cent survival and 60 per cent survival^10–14^. In the UK it is widely accepted that a mortality risk of more than 5 per cent according to NELA predictive score is a high risk of mortality and triggers targets such as consultant-delivered care and postoperative admission to critical care. However, there is no further qualification of what quantifiable risk is ‘very’ high or ‘extremely’ high, or even ‘prohibitively’ high to aid determination of futility^15^.

In contrast, qualitative futility refers to an intervention that, even if successful, will not result in acceptable functional status or quality of life for a patient (principles 3 and 6)^8^. The medical profession is increasingly recognizing the importance of quality of life outcomes, and are building them into outcome measures in clinical trials alongside traditional outcomes such as mortality and length of hospital stay^16^. Quality of life outcomes are challenging to delineate, as what one person may deem as good quality of life may be unacceptable to another; clinicians have been shown to underestimate patient-reported quality of life^17^; and high-risk patients have been shown vastly to underestimate their risk of morbidity, serious complications, and risk of discharge to nursing facilities alongside their risk of mortality^18^.

With the complexities in defining and applying the principles of futility, very little of the discourse has centred around the large, high-risk emergency surgical population. This scoping review aimed to identify and analyse the published work around surgical futility as it relates to emergency laparotomy.

## Methods

### Research question, search strategy, and selection criteria

A research team was established to advise on the broad question to be asked, search terms to be used, and databases to be searched. All researchers had experience in emergency laparotomy research and audit, and were engaged in emergency laparotomy decision-making in their clinical practice. Using the framework by Arksey and O’Malley^19^, this scoping review contained five key phases: forming the research question; identifying relevant studies; study selection; data charting; and collating, summarizing, and reporting the results.

The research question for this study was ‘What are the applied definitions of surgical futility in the emergency laparotomy setting and what outcomes were reported?’ A search of the Cochrane Library, Google Scholar MEDLINE, and Embase was done up to 1 November 2021 using the search terms ‘futile’, ‘futility’, and ‘non-beneficial’ paired with either ‘treatment’, ‘surgery’, ‘laparotomy’, or ‘operation’. The search was performed by H.J.E.; title and abstracts were screened and adjudicated where necessary by S.J.M. The full search term strategy is listed in *Appendix S1*. No time limits were attached to the search, but results were limited to the English language only. Publications pertaining to futility in non-surgical care and surgery other than emergency laparotomy were excluded. The results were reported according to the PRISMA extension for scoping reviews, and the PRISMA-SCR checklist can be found in *Appendix S2*^20^.

Articles were eligible for inclusion if their study population was patients undergoing emergency laparotomy for any reason other than trauma and outcomes included predictors of futility. For the purposes of this scoping review, all definitions of futility were included.

Data points extracted and charted from the studies comprised of study definition of surgical futility, how this definition was determined, the study primary outcome, details of the surgical population included, number of patients included, single or multicentre, risk prediction method used, and predictors of surgical futility. No additional data were required from the authors of the included papers. Results were compiled as a narrative synthesis.

Documents within the grey literature (conference presentations, opinion pieces, etc.) were not included in this review, but were used to provide context in both the ‘Introduction’ and ‘Discussion’ sections.

## Results

**Fig. 2:**
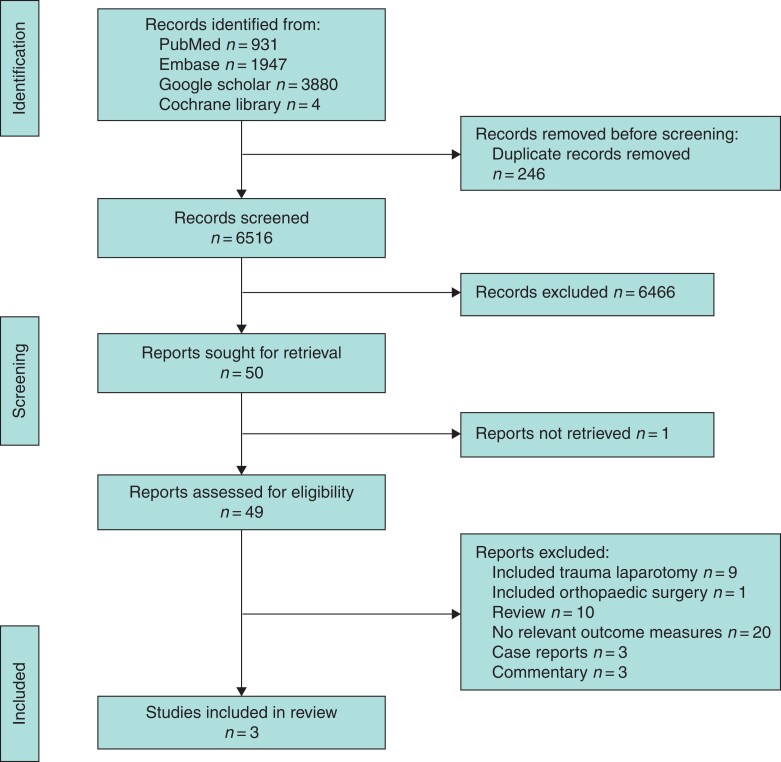
PRISMA 2020 flow diagram of article identification

A total of 6762 articles were screened, with three studies meeting the inclusion criteria (*Fig. 2*). All three studies identified in this scoping review were retrospective cohort studies^21–23^. A total of 105 157 patients were included, 1114 of whom were part of the ‘futility’ cohort (*Table 1*). Two of the studies were based in North America and one originated in the UK^21–23^. All were published from 2019 onwards^21–23^. The primary outcome of all three studies were clinical and patient predictors of futile surgery^21–23^.

**Table 1:** A comparison of the studies into ‘surgical futility’

	Source of futility definition	Surgical Population	Number of patients		Single or multi centre	Risk prediction method	Primary outcome	Predictors of ‘surgical futility’
			Total	Futility Cohort				
**Chiu *et al*. 2019**	Local surgeon survey Survival histogram	5 procedures: Colectomy Small bowel resection Adhesiolysis Control of bleeding ulcer Exploratory laparotomy	92 556	567	Multi-centre (NSQIP database)*	NSQIP score	Predictors of early death in extreme risk patients	Age >80 Partially or fully dependent for personal care Sepsis on admission Ventilator dependent in the first 48 hours
**Aggarwal *et al*. 2020**	Survival histogram	All non-trauma emergency laparotomy patients	13 953	519	Multi-centre (ELC database)^†^	P-POSSUM score	Predictors of early death in all patients	Age Log serum creatinine Log P-POSSUM score Bowel ischaemia Small bowel perforation
**Kao *et al*. 2020**	Survival histogram	All non-trauma emergency laparotomy patients	534	28	Single centre	APACHE-II score	Predictors of early death in all patients	Age Serum creatinine Glasgow coma score Arterial lactate Blood pH

* National Surgical Quality Improvement Program (American College of Surgeons). ^†^Emergency Laparotomy Quality Improvement Collaborative (South of England).

All three of the identified studies used the principle of quantitative futility, applying time to death: ‘early postoperative death’^21–23^. What was defined as an early postoperative death varied between a death up to 48 and 72 hours after the procedure. Justification for these timelines included that death during the first 48 hours may be attributable to physiological derangement caused by the surgical pathology, rather than an operative complication^21,23^; the patient’s life was not ‘meaningfully prolonged’ by the surgical procedure^21,23^; and allowing for re-look operations, which are usually carried out within 48 to 72 hours of the initial procedure^21^. Finally, when patient survival was graphed by postoperative days there was a consistent trend of 40 per cent of deaths occurring in the first 72 hours^21–23^.

Chiu and colleagues approached the issue by examining patients who had a higher predicted risk of dying from the procedure than surviving. Patients were identified from the NSQIP database undergoing one of five emergency general surgical conditions (*Table 1*) were ‘extreme risk patients’ and had a predicted 30-day mortality of more than 75 per cent^21^. This threshold was decided on following departmental discussions at their institution with colleagues about what mortality risk threshold they would deem too risky to offer an operation^21^. The primary outcome was death within 48 hours of the procedure (what they defined as futile surgery) and secondary outcomes were discharge destination and morbidity, which they defined as any postoperative complication, as proxy quality of life markers. Their analysis included 1794 ‘extreme risk patients’, which represented 1.9 per cent of the total number of emergency laparotomy patients in the database in the period examined^21^. By their definitions, futile surgery occurred in 567 ‘extreme risk patients’ (31.6 per cent), eight times higher than in the patients with a mortality risk of less than 75 per cent^21^. Predictors of futile surgery are listed in *Table 1*. Chiu *et al*. noted a decline in the proportion of extreme risk patients being operated upon during the 9 years of the study: from 2.4 per cent to 1.5 per cent of patients having an emergency laparotomy^21^. In terms of morbidity, extreme risk patients had more than double the respiratory complications, three times more cardiac complications, and 3.5 times more renal complications than their lower-risk counterparts^21^. Only 5.5 per cent of extreme risk group survivors who lived independently preoperatively were discharged back to their own home, compared to 63.7 per cent of the lower-risk group^21^.

In contrast to applying an established risk score like NSQIP, Kao *et al*. analysed the frequency of their early postoperative deaths and then sought to create a new risk score. This group defined early postoperative death as within 72 hours of procedure^23^. They included 28 patients in their futility cohort, which made up 5.3 per cent of their overall emergency laparotomy patient group^23^. Patients in the early postoperative death group most commonly presented with bowel ischaemia (65.4 per cent), whereas obstruction and pneumoperitoneum were more common pathologies in patients that survived the initial postoperative period^23^. Age, Glasgow Coma Scale, blood pH, serum creatinine, and arterial lactate were independent predictors of futility and were used to create the CELIOtomy score to guide shared decision-making discussions^23^. This score was internally cross-validated but not validated externally^23^.

The remaining study sought to identify what leads to early deaths in futile patients, and whether it is patient-related factors or organizational factors that contribute. Aggarwal *et al*. examined more than 13 000 patients from a multicentre database and identified 519 (4 per cent) who died in the first 72 hours after emergency laparotomy, then compared their presenting characteristics to all other patients in the database^22^. Predictors of early death were age, log P-POSSUM score, log serum creatinine, bowel ischaemia, or small bowel perforation^22^. Patients in the early death cohort were more likely to have both a consultant anaesthetist and consultant surgeon in theatre, goal-directed fluid therapy, and be admitted to critical care postoperatively, but were less likely to reach theatre in a timely fashion^22^.

## Discussion

This scoping review has reported on the exploration of futility in the setting of emergency laparotomy. Despite being one of the largest and high-risk patient populations in the UK, only three publications were found, although all were recent potentially highlighting a move towards exploring this difficult topic. All these groups applied quantitative futility principles using mortality as their outcome marker and supplementing that with a timeline under the umbrella term ‘early postoperative death’^21–23^.

While using mortality is an objective and recognized outcome marker, this binary approach has two limitations. All studies defined futility according to relevant clinical reasons guided by the surgeons involved^21–23^. Indeed, few surgeons would want to operate on a patient that is going to die within 48–72 hours. However, surgeons know that high-risk of an early postoperative death is exactly that, a high risk and not an absolute certainty. It is possible that future research will lead to the development of new operative or medical approaches for high-risk patients and surgeons would not want to deny high-risk groups, such as those with ischaemic bowel, that opportunity. The development of a new score to predict futility (CELIOtomy) appears to have the advantage over the application of established scores that have been developed for 30-day mortality, not early postoperative death^22^. This score needs validation within another cohort. In the future, both a futile risk score and the NELA 30-day score could both be calculated preoperatively to provide greater information for decision-making.

The second limitation of previous work is that it overlooks qualitative futility^22,23^. Patients and relatives have reported feeling that mortality was a particular focus of clinicians during the decision-making process and although the risk of death is important, patients have reported that returning home and not having formation of a stoma are greater concerns^24,25^. Chui *et al*. explored morbidity and return to independence within their work, reporting that extreme high risk patients have greater morbidity and increased dependence than a lower-risk patient group^21^. These findings begin to address knowledge gaps about postoperative recovery in risk patients and although not specifically addressing futility, do encompass the principles of what is important to the patient should they recover (principles 3 and 6). The application of a futility score alongside a 30-day morality score opens the opportunity for accurate prediction of morbidity to enhance decision-making discussions. Currently, the only score validated for 30-day morbidity, mortality, and independence at discharge is the Clinical Frailty Score^26,27^, but prediction of futility has not been analysed.

For patients who have an early postoperative death, the hours in the perioperative period are extremely difficult for both the patient and their families: consciousness may not recover; they might have delirium; pain-management issues; cardiovascular support; or care may be in a critical care setting. These factors have been shown to increase anxiety, complex grief, and depressive episodes in relatives^28^. The role of palliative care *versus* optimal perioperative care is a difficult balance to achieve in this setting of unknown outcome^29,30^. Research exploring this could improve the sensitive preoperative discussions around decision-making.

Surgical futility is an emotive term and concept. Some clinicians feel the terminology has a negative connotation and have suggested ‘non-beneficial’ or ‘limited-benefit’ care, or ‘medically inappropriate’ as alternatives^31,32^. In addition, there is no documented patient and public involvement work exploring the perception of ‘futility’. Critics argue that the underlying goal of this conversation is to agree upon a definition that can be used for clinicians to determine when a treatment can be ‘unilaterally withheld’, even over the objections of a competent patient’^12,33^. In this way, futility is also inexorably linked with the ethical principle of patient autonomy: the patient’s right to self-determination; the right to choose or even refuse treatment. There is often a feeling that futility is an adversarial and contentious issue between patients and clinicians^12,33,34^. This is in contrast to a body of evidence that shows that clinicians care deeply about preventing unnecessary harm to their patients, and that clinicians who provide a high level of what they perceive to be inappropriate or ‘futile’ care suffer from significant levels of emotional distress, depression, and burnout^35–37^.

The intersection of clinician decision-making, respect of patient autonomy, and patient-led treatment goals is patient-centred shared decision-making and involves discussions of patients’ values. One solution to improving these discussions and preventing non-beneficial surgery is improving communications skills. Simulation-based clinical communications scenarios and communications frameworks can significantly improve clinicians’ confidence in having patient-centred goals of care discussions^38–40^. Alternatively, or in addition, application of the BRAN framework allows the key points of a decision-making conversation to be presented in a bitesize fashion, and signposts the conversation in a way that is easier for the multidisciplinary team to deliver under pressure, easier to digest for patients and relatives in acutely stressful situations, and empowers the clinicians to discuss the option of changing the focus of active management to non-operative^41^.

All three futility studies focused on patients undergoing emergency laparotomy^21–23^. To provide greater insight into futility requires definition of the denominator: those patients that require emergency laparotomy but do not undergo such. To date, there has only been one prospective single centre study, which demonstrated that 32 per cent (100 of 314) of consecutive patients presenting to acute surgical services did not undergo emergency laparotomy^42^. A third were alive after 30 days and had better renal function, albumin, and lactate levels on admission^42^. The main reason documented for not operating was ‘not fit enough for surgery’^42^. An upcoming multicentre observational study will attempt to define this patient group, analyse their outcomes, and compare the patients to a group who had emergency surgery^43^.

There remains a paucity of research defining, exploring, and analysing futile surgery in patients undergoing emergency laparotomy. With limited published work focusing on quantitative futility and the binary outcome of death, research is urgently needed to explore all principles of futility, including the wishes of patients and their families.

## Supplementary Material

zrac023_Supplementary_Data

## Data Availability

No new data were generated from this work with all peer-reviewed publications available online.
