# Supplementary material for: The false dichotomy of surgical futility in the emergency laparotomy setting: scoping review
Source: BJS Open. 2022 Apr 7;6(2):zrac023. doi: 10.1093/bjsopen/zrac023 (PMC8988868; doi:10.1093/bjsopen/zrac023)
Supplement: zrac023_Supplementary_Data [file zrac023_supplementary_data.zip › Supplementary_Appendix_1.docx]

**Appendix 1**

Full search strategy used.

(futil* AND surgery) OR (futil* AND operation) OR (futil* AND treatment) OR (non-beneficial AND surgery) OR (non-beneficial AND operation) OR (non-beneficial AND treatment)
